# Quantifying the global burden of mental disorders and their economic value

**DOI:** 10.1016/j.eclinm.2022.101675

**Published:** 2022-09-28

**Authors:** Daniel Arias, Shekhar Saxena, Stéphane Verguet

**Affiliations:** Department of Global Health and Population; Harvard T.H. Chan School of Public Health; 677 Huntington Ave., Building 1, 12th Floor; Boston, MA 02115, United States

**Keywords:** Mental health, Disease burden, Premature mortality, Economic burden

## Abstract

**Background:**

Epidemiological and economic estimates suggest that the global burden of mental disorders is considerable, both in its impacts on human health and losses to societal welfare. The availability of additional data and the emergence of new approaches present an opportunity to examine these estimates, which form a critical part in making the investment case for global mental health.

**Methods:**

This study reviews, develops, and incorporates new estimates and methods in quantifying the global burden of mental illness. Using a composite estimation approach that accounts for premature mortality due to mental disorders and additional sources of morbidity and applying a value of a statistical life approach to economic valuation, we determine global and regional estimates of the economic cost that can be associated with mental disorders, building on data from the 2019 Global Burden of Disease study.

**Findings:**

We estimate that 418 million disability-adjusted life years (DALYs) could be attributable to mental disorders in 2019 (16% of global DALYs)—a more than three-fold increase compared to conventional estimates. The economic value associated with this burden is estimated at about USD 5 trillion. At a regional level, the losses could account for between 4% of gross domestic product in Eastern sub-Saharan Africa and 8% in High-income North America.

**Interpretation:**

The burden of mental illness in terms of both health and economic losses may be much higher than previously assessed.

**Funding:**

None.

**Research in context**

**Evidence before this study**

Prior work has established that mental disorders are significant causes of disability, important risk factors for premature mortality, and costly with respect to societal welfare. With respect to the epidemiological burden of mental disorders, the Global Burden of Disease study attributes nearly 15% of years of life lost to mental disorders, making mental illnesses one of the largest causes of disability worldwide. In terms of economic costs, global evaluations by Bloom and colleagues estimated that the value of losses due to mental disorders was roughly 1·3 trillion US dollars (USD) in 2010 ($1·6 trillion in 2019) when disability-adjusted life years (DALYs) were valued at one times gross domestic product (GDP) per capita. The authors projected that these losses would grow to nearly 2·5 trillion USD 2010 (or approximately 3 trillion USD in 2019) by 2030. While these findings are striking, work by Vigo and colleagues argues that the global burden of mental disorders is itself underestimated—and that a more complete picture of the burden would encompass morbidity and mortality due to dementias, epilepsy, migraine, tension-type headache, and self-harm, along with a third of the burden of musculoskeletal disorders, to account for somatoform and chronic pain disorders.

**Added value of this study**

Our study expands the estimation of the global burden of mental disorders and of its associated economic value. We build on previous estimation approaches by capturing premature mortality due to mental disorders using pooled risk ratios of mortality from a systematic review of mental disorders to determine the population attributable fraction (PAF) of premature mortality. Inclusion of premature mortality through the PAF reflects the dual status of mental illness as a risk factor for death and a direct cause of disability. Using this inclusive estimate, we apply monetary values per DALY to reach estimates of the global economic value of the mental burden of disease.

**Implications of all the available evidence**

Our findings suggest that both the epidemiological and economic burden of mental disorders may be larger than previously estimated, and that underestimation may be larger among regions where premature mortality due to mental disorders is greater. Combined with previous studies, our work contributes to ongoing discourse on the measurement and aggregation of the global burden of mental disorders and underscores the considerable magnitude of health and welfare loses associated with mental illness. This has implications for global and national policies on mental health and highlights the need for additional investments in this area.

Alt-text: Unlabelled box

## Introduction

Mental health is an essential part of human flourishing. As defined by the World Health Organization (WHO), it encompasses “a state of well-being in which every individual realizes [their] own potential, can cope with the normal stresses of life, can work productively and fruitfully, and is able to make a contribution to [their] community.”^1^ For much of the global population, however, attaining this state of mental health is an enduring challenge, with over one billion people worldwide living with a mental or addictive disorder.^2^ Mental disorders are both leading causes of disability and significant risk factors for premature mortality.^2^^,^^3^ At all levels of sociodemographic development, this burden of morbidity and mortality is rising.^4^ Furthermore, as the COVID-19 pandemic continues, there is growing and alarming evidence of its detrimental psychological and psychiatric effects—for patients, health care workers, and the public overall.^5^

The magnitude of disability caused by mental disorders has galvanized a global movement and a call to action for greater investment and prioritization for mental health.^6^ This movement has emphasized the importance of investing in mental health as a means of promoting sustainable development, human rights, and social inclusion.^4^

A critical link between mental health and development arises from the economic consequences of mental disorders. A growing body of literature suggests that mental disorders are costly, both in the direct medical costs of care, outpatient visits, and hospitalizations, and in indirect costs, such as losses in income and productivity due to disability, which may cause absenteeism and presenteeism.^7^ These costs further worsen conditions of poverty^8^—a vulnerability that, in turn, worsens mental health, feeding a vicious cycle of poverty and illness.^9^ At the national level, mental disorders deplete the supply of labor and capital, resulting in poorer economic output.^10^ Among households and nations alike, the burden of mental illness thus has considerable economic consequences and poses a challenge to both health and wealth.

Evaluating the economic burden of mental illness is a critical part in making the investment case for global mental health, informing public health decision-making, and guiding priority-setting and the scale up of much-needed interventions.^11^ At the global level, however, the most recent estimate of the economic impact of mental disorders was published in 2011, using burden of disease estimates from 2004.^12^ This study used three distinct approaches to quantify the economic burden of non-communicable diseases (NCDs), including mental illnesses.^12^ The first is a cost-of-illness (COI) analysis, which includes the direct costs of illness as well as the indirect costs (e.g., lost productivity). The second is a value of lost output (VLO) approach, which estimates the effects of illness on gross domestic product (GDP) due to the depletion of labor and capital. The third builds from value of a statistical life (VSL) approaches and attempts to capture a population's willingness to pay to reduce morbidity and mortality associated with illness. This expands on the COI and VLO approaches, as it puts an economic value on the loss of health itself.

With a third of disability-adjusted life years (DALYs) due to NCDs arising from mental disorders, this landmark paper estimated that the value of losses due to mental disorders was roughly 1·3 trillion USD in 2010 (1·6 trillion USD in 2019) when DALYs were valued at one times GDP per capita.^12^ The authors further projected that these losses would grow to nearly 2·5 trillion USD 2010 (or approximately 3·0 trillion USD in 2019) by 2030. (See Supplementary appendix Table S1 for estimates from the other two approaches and Table S2 for estimates by World Bank income group.) These estimates have been widely cited in calls to action concerning global mental health.^4^^,^^13^

While the estimates presented from this paper remain staggering and salient, new studies estimating the morbidity and mortality associated with mental illness have since become available.^14^, ^15^, ^16^ These studies suggest that previous (and current) estimates of the global burden of mental disorders may be considerably underestimated, which, in turn, has implications for estimating the true economic burden of mental illness.

The most recent estimates of morbidity and mortality due to mental disorders come from the Global Burden of Disease (GBD) 2019 study.^17^ The GBD study provides disease burden estimates using DALYs, years of life lost (YLLs), and years lived with disability (YLDs), which are then aggregated within a hierarchical grouping scheme that classifies causes of disability and death at different levels of mutually exclusive and completely exhaustive categories. (Mental disorders are a Level 2 condition, nested under NCDs; see Table S3.)

While GBD remains the gold standard for global epidemiologic estimation, the nature of the GBD scheme—in particular, the rationale for grouping certain conditions under mental disorders or not—has been the subject of debate in the literature.^14^^,^^18^^,^^19^ In particular, work by Vigo et al. (2016) published in *The Lancet Psychiatry* argues for an expanded classification of mental disorders under the GBD classification scheme to account for underestimation of the burden of mental disorders.^14^ The authors attribute this underestimation to five main causes: 1) the distinction drawn between mental and neurological diseases; 2) the categorization of self-harm and suicide under injuries; 3) the classification of all chronic pain and somatoform disorders under musculoskeletal disorders; 4) the exclusion of personality disorders; and 5) the exclusion of premature mortality due to mental disorders. Using data from the 2013 GBD study, Vigo and colleagues re-allocated the entire burden of dementias, epilepsy, migraine, tension-type headache, and self-harm to mental disorders. In addition, a third of the burden of musculoskeletal disorders without anatomical correlate (i.e., somatoform disorders with prominent pain) was attributed to mental disorders.^14^ This reallocation attributed 13% of DALYs to mental disorders, a 6 percentage point increase from the GBD estimate of 7%.

In this paper, we attempt to revisit the estimation of the global burden of mental disorders and of its associated economic value. Our aim is to characterize potential underestimation of the burden of mental disorders and to quantify the economic value of this burden under different estimation approaches. Specifically, we expand on Vigo et al.^14^ by capturing premature mortality due to mental disorders using pooled risk ratios of mortality from a systematic review of mental disorders^15^ to determine the population attributable fraction (PAF) of premature mortality. Inclusion of premature mortality through the PAF presents a novel composite approach that can more broadly capture attributable morbidity and mortality. Using this approach on GBD 2019 estimates, we then apply monetary values to DALYs to reach estimates of the global economic value of the mental burden of disease using a VSL approach. The VSL approach—in contrast to COI and VLO approaches—includes an economic valuation of mortality risk reductions in monetary terms, and thus enables comparison across sectors (beyond the sole health sector) that can motivate decision-making toward ameliorating welfare and societal mental health. Our findings suggest that both the epidemiological and economic burden of mental disorders could be larger than previously estimated, and that underestimation would be larger among regions where premature mortality due to mental disorders is greater.

## Methods

To estimate the economic burden of mental disorders, we first estimate the attributable mental burden of DALYs under various estimation approaches using data from the 2019 GBD study (available from the Global Health Data Exchange at https://ghdx.healthdata.org/gbd-2019). Second, we apply a monetary value to a DALY to yield an economic assessment associated with these burden estimates.

### Burden of mental disorders

In our analysis, we replicate the approach of Vigo et al. (2016) using GBD 2019 estimates, applying a similar re-allocation formula to YLLs, YLDs, DALYs, and deaths. Our approach, however, differs in some key respects.

First, we agree with Whiteford and colleagues in viewing the assigning of the entire burden of suicide and self-harm to mental disorders as an overestimate, and consequently do not reallocate all DALYs due to suicide towards the mental health burden.^18^ While it is empirically clear that mental disorders elevate the risk of death by suicide and that the majority of suicides appear to be due to mental disorders,^20^ we view assigning the entirety of this burden to mental disorders as overinclusive, which we avoid to favor a conservative estimation strategy.

Second, we attempt to capture premature mortality attributable to mental disorders, recognizing that persons with mental disorders are at elevated risk of all-cause mortality,^15^ unnatural death,^21^ and deaths due to natural causes.^22^ Not capturing this share of mortality is likely to be a prominent cause of underestimating the burden of mental illness, particularly in countries where the dominant share of the DALY burden is mortality (rather than morbidity).

Following Vigo and colleagues, we replicate reallocations in neurological and musculoskeletal conditions, and further include alcohol and mental use disorders, as these were previously classified under mental disorders within the GBD classification.

This provides estimates of YLDs due to mental disorders. We then estimate the PAF of mortality due to mental disorders, using GBD prevalence estimates and relative risk estimates for natural-cause and unnatural-cause mortality generated from a systematic review and meta-analysis by Walker et al.^15^ A comparison of our allocation approach with those of Vigo et al. and the original GBD hierarchical allocation is shown in Table 1.

**Table 1:** Comparison of the composite allocation approach with the reallocation approach and with the original Global Burden of Disease (GBD) hierarchical allocation, with respect to the burden of mental disorders.

	1. Original allocation	2. Reallocation approach^14^	3. Composite approach
**Schizophrenia**	Yes	Yes	Yes
**Depressive, bipolar, anxiety disorders**	Yes	Yes	Yes
**Eating disorders**	Yes	Yes	Yes
**Autism spectrum, AD(H), and conduct, disorders**	Yes	Yes	Yes
**Substance abuse disorders**	Included in GBD 2016; classified separately since GBD 2017	Yes (with additional deaths due to alcohol use included in Vigo et al. 2020)^31^	Yes
**Neurological disorders**	No	Yes	Yes
**Chronic pain syndrome and somatoform pain disorders**	No	Yes, 33% of DALYs	Yes, 33%
**Self-harm / suicide**	No	Yes, all DALYs	Yes, % of YLLs due to unnatural death based on PAF
**Premature mortality due to mental disorders**	No	No	Non-communicable diseases: Yes, % of YLLs due to natural death based on PAF
			Infectious, maternal, and neonatal diseases: No

*Yes: causes of morbidity and mortality included in the burden of mental disorders. AD(H): attention deficit (and hyperactivity). DALY: disability-adjusted life year. YLL: years of life lost. PAF: population attributable fraction.

Our approach to capturing premature mortality relies on a pooled relative risk estimate for mortality by natural and unnatural causes, drawn from 148 studies identified by Walker et al.^15^ These studies collectively reflect over 338,000 deaths across 29 countries and 6 continents. The majority of deaths (67%) recorded in studies with disaggregated data arose from acute and chronic illnesses, while unnatural causes such as injury and suicide represented 18% of deaths (the rest being unallocated). Overall, the pooled risk of all-cause mortality was 2·2 times higher (95% confidence interval (CI): 2·1-2·3) among people with mental disorders compared to those without. Using this relative risk estimate, Walker and colleagues calculated a PAF to estimate that 8 million deaths were due to mental disorders in 2012.

While Walker and colleagues used a global estimate of the worldwide prevalence of mental disorders in their study to calculate the PAF, we use GBD estimates of prevalence to derive both global- and country-level results. The PAF for a given disorder *d* and country *c* is given by:

(1) $$PAF_{d,c}=\frac{p_{d,c}\left(RR_{d}-1\right)}{1+p_{d,c}\left(RR_{d}-1\right)},$$

where $p_{d,c}$ is the prevalence of a given disorder in a country and $RR_{d}$ is the relative risk of mortality estimated by Walker et al.^15^

We separately estimate the PAF for natural and unnatural causes of mortality. Using the calculated PAF estimates, we estimate YLLs attributable to mental disorders by multiplying the PAF by the national burden of mortality. For natural causes of death, we conservatively apply the PAF against YLLs attributable to NCDs. For unnatural causes of death, we apply the PAF against YLLs due to self-harm and injuries. These YLLs are then combined with the YLDs calculated previously to provide DALYs.

### Economic burden of disease

To estimate the economic cost associated with premature mortality and morbidity tied to mental illnesses, we assigned a monetary value to attributable DALYs. VSL approaches assign a monetary value to small reductions in mortality risks.^23^ Drawing from these approaches, Jamison and colleagues have estimated monetary values of statistical life years,^24^ which Khadka and colleagues recently adapted to quantify the economic value of changing mortality risk by cause of death in low- and middle-income countries (LMICs).^25^ While VSL approaches are not meant to assign monetary values to full life years or years lived with illness or disability,^23^ the Copenhagen Consensus has previously implemented the use of GDP per capita as a proxy for the monetary value of a DALY as a standard estimate.^26^ Values of one and three times GDP per capita have been suggested as proxies for the value of a DALY.^27^^,^^28^ Estimates of $1,000 and $5,000 per DALY have also been used, with the justification that these would be reasonable and convenient lower and upper values, particularly for low-income and lower-middle income countries.^29^^,^^30^

Consistent with previous approaches, we use GDP per capita (USD 2019) for our base-case value of a DALY. GDP inputs are reported in 2019 USD and obtained from the World Bank's World Development Indicators; for consistency with our epidemiological inputs, we convert to per capita values using GBD population estimates.

### Sensitivity analyses

The primary focus of this paper concerns structural uncertainty in determining the burden of mental illness, resulting in the evaluation of three different estimation approaches. To address parameter uncertainty within each approach, we apply a three-way sensitivity analysis. First, following a simple intuitive approach, we incorporate the upper and lower uncertainty intervals (UIs) provided by GBD 2019 for YLLs, YLDs, and DALYs to account for parameter uncertainty. Second, we use the upper and lower values of prevalence estimates and of the 95% confidence intervals (CIs) of the pooled relative risk of all-cause mortality from Walker and colleagues in our composite approach.^15^ Third, our lower bound estimates are set to reallocate one sixth of the burden of musculoskeletal disorders proposed by Vigo and colleagues,^14^ while our upper bound estimates are set to reallocate one half of this burden.

In addition to our base-case economic valuation, we further report our VSL estimates using three times GDP per capita as the value of a DALY. (Alternative valuations using values of $1,000 and $5,000, as well as purchasing power parity (PPP)-adjusted GDP per capita, are reported in Tables S5 and S6 of the Supplementary appendix.)

### Statistical analysis

All computations were conducted using R software (version 3·6·2).

### Ethics statement

The research draws exclusively on secondary data reported at the national or subnational level. As such, it does not involve data collection, experimentation, or investigation concerning human subjects. The Institutional Review Board (IRB) of the Harvard T.H. Chan School of Public Health has determined that the study was not human subjects research and that additional review was not required (protocol # IRB20-1946, determined on November 13, 2020).

### Role of funding source

This study received no funding. All authors (DA, SV, and SS) had access to the data and shared the decision to submit this article for publication.

## Results

Under GBD 2019, over 125 million DALYs were attributed to mental disorders, or roughly 5% of the global burden. After including alcohol and drug use, neurological disorders, chronic pain, suicide, and self-harm, the share due to mental disorders rose to 12% of global DALYs (approximately 321 million DALYs). Under the composite approach, an additional 97 million DALYs were attributed to mental disorders, encompassing, in total, over 16% of global DALYs. Under all three methods, the burden of mental disorders (in DALYs) exhibited a country-income gradient, with mental disorders comprising over twice the burden of disease in high-income countries compared to low-income countries.

**Figure 1:**
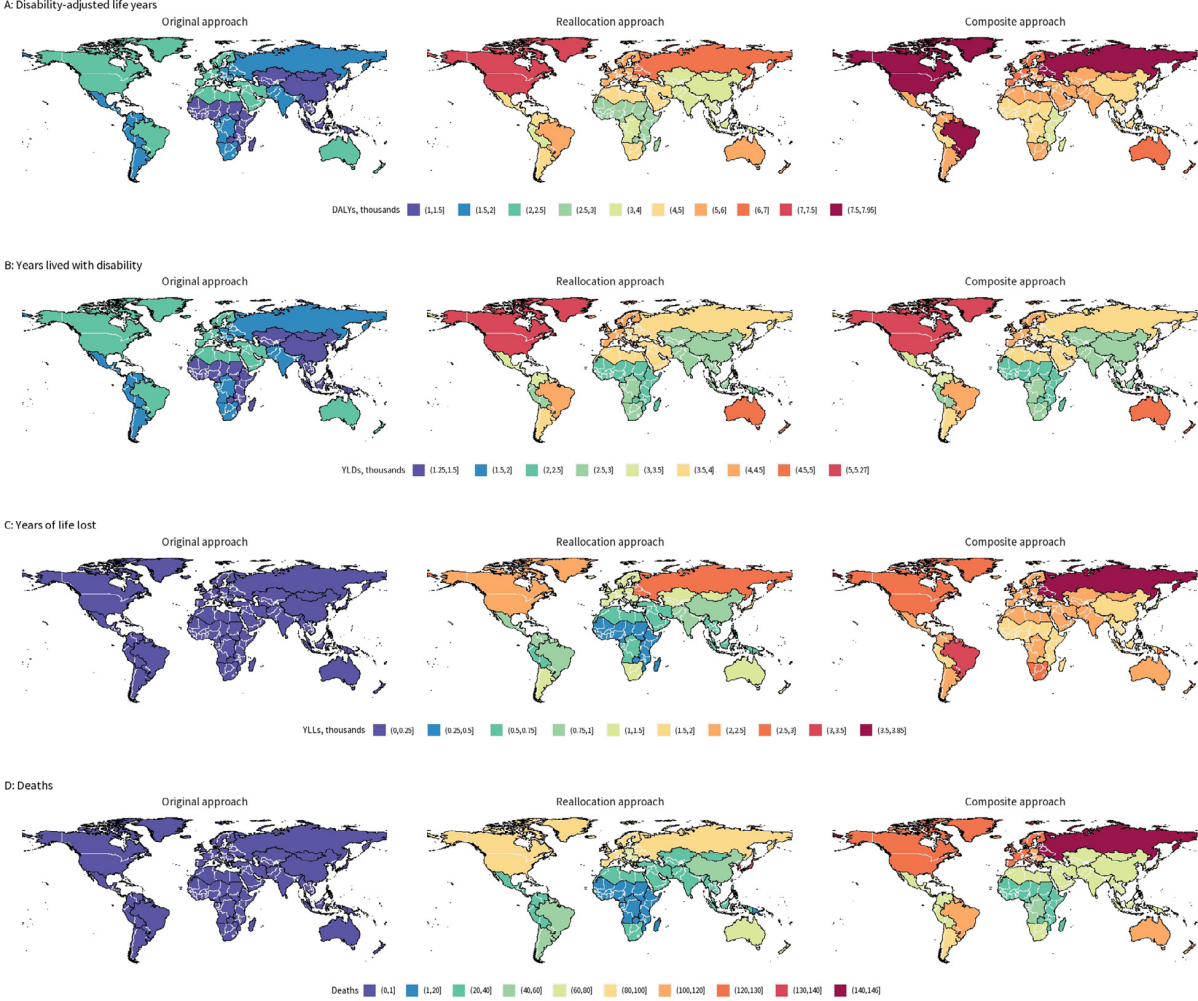
DALYs, YLDs, YLLs, and deaths attributable to mental disorders in 2019, by estimation approach, per 100,000 population. Values are aggregated by GBD region. DALYs: Disability-adjusted life years; YLDs: years lived with disability; YLLs: years of life lost; GBD: Global Burden of Disease.

Rates of DALYs, YLDs, YLLs, and deaths attributable to mental disorders under the different estimation approaches are presented by GBD region (Figure 1). Geographically, the composite approach allocated a large portion of DALYs (to mental disorders) in Eastern Europe, North and Latin America, and sub-Saharan Africa. This is largely driven by the inclusion of premature mortality in the composite approach. Estimates of attributable DALYs by country income group and GBD region are reported in Table 2. (Estimates of attributable YLDs, YLLs, and deaths are reported in Table S4-6.)

**Table 2:** Disability-adjusted life years (DALYs) attributable to mental disorders as totals (millions) and percentages of overall burden, by World Bank income group classification and GBD region, under three estimation approaches.

	Original approach						Reallocation approach						Composite approach					
	DALYs			% of burden			DALYs			% of burden			DALYs			% of burden		
	Estimate	Lower bound	Upper bound	Estimate	Lower bound	Upper bound	Estimate	Lower bound	Upper bound	Estimate	Lower bound	Upper bound	Estimate	Lower bound	Upper bound	Estimate	Lower bound	Upper bound
Global	125·3	93·0	163·2	4·9	4·1	5·8	321·2	198·6	505·2	12·7	8·7	18·0	417·7	276·7	608·4	16·5	12·1	21·6
High income	24·5	18·1	32·0	6·7	5·7	7·6	74·4	46·2	117·9	20·3	14·4	28·1	81·0	52·8	120·3	22·1	16·5	28·7
Upper-middle income	45·6	33·8	59·7	5·6	4·7	6·4	117·5	69·5	191·6	14·3	9·7	20·5	156·6	99·3	236·2	19·1	13·9	25·3
Lower-middle income	44·9	33·0	58·9	4·3	3·6	4·9	108·8	65·0	174·1	10·4	7·1	14·6	147·4	90·2	225·7	14·1	9·8	18·9
Low income	10·1	7·4	13·5	3·4	2·9	3·8	20·3	12·0	32·6	6·7	4·6	9·3	32·3	19·8	49·9	10·7	7·7	14·2
East Asia	21·0	15·7	27·3	5·3	4·5	6·0	55·8	32·8	91·8	14·1	9·5	20·3	72·7	45·6	109·7	18·3	13·2	24·3
Southeast Asia	9·1	6·7	11·9	4·6	3·9	5·3	23·3	12·4	40·4	11·8	7·2	17·8	32·3	18·8	52·0	16·3	11·0	22·9
Oceania	0·2	0·1	0·2	3·0	2·7	3·3	0·4	0·2	0·6	7·1	4·7	9·8	0·6	0·4	1·0	12·2	8·6	16·2
Central Asia	1·3	0·9	1·7	4·4	3·7	5·1	3·7	2·3	5·8	13·1	9·3	17·8	4·7	2·9	7·2	16·4	11·8	22·0
Eastern Europe	3·5	2·6	4·5	3·9	3·3	4·5	13·4	9·0	20·2	15·1	11·5	20·0	16·1	10·7	23·5	18·1	13·6	23·3
Central Europe	1·7	1·3	2·3	4·3	3·7	4·8	5·7	3·4	9·4	14·1	10·0	19·9	6·8	4·2	10·5	16·8	12·2	22·3
Caribbean	0·8	0·6	1·0	5·2	4·5	5·8	1·7	1·0	2·8	11·7	8·2	16·0	2·7	1·7	4·1	18·0	13·3	23·5
Central Latin America	4·1	3·0	5·5	6·2	5·3	7·0	10·1	5·9	16·4	15·1	10·4	21·0	14·2	8·9	21·8	21·3	15·5	27·9
Tropical Latin America	5·1	3·7	6·6	7·5	6·2	8·8	11·6	7·0	18·6	17·3	11·7	24·7	16·9	11·7	24·1	25·2	19·5	32·1
Andean Latin America	1·1	0·8	1·4	7·0	6·1	7·8	2·3	1·4	3·8	15·0	10·5	20·3	3·2	1·9	4·9	20·4	14·9	26·7
North Africa and Middle East	10·7	7·8	14·1	8·0	6·9	9·0	21·5	12·3	35·3	16·0	10·8	22·5	31·3	19·3	48·1	23·4	17·0	30·7
Southern Sub-Saharan Africa	1·2	0·9	1·6	3·2	2·6	3·8	3·2	2·0	4·9	8·3	5·7	11·6	4·7	3·1	6·8	12·3	9·0	16·1
Western Sub-Saharan Africa	6·7	4·9	9·0	2·6	2·2	2·9	14·7	8·2	24·5	5·6	3·6	8·0	20·8	11·9	33·5	7·9	5·3	10·9
Central Sub-Saharan Africa	2·1	1·5	2·8	3·7	3·1	4·2	4·1	2·4	6·6	7·2	4·9	9·9	6·4	3·8	9·9	11·0	7·7	14·8
Eastern Sub-Saharan Africa	5·8	4·2	7·7	3·5	2·9	4·0	11·3	6·9	17·8	6·8	4·8	9·2	15·8	9·9	24·2	9·5	6·8	12·6
South Asia	28·8	21·2	37·6	4·6	3·8	5·3	70·8	43·3	111·1	11·2	7·7	15·6	96·0	59·9	144·2	15·2	10·7	20·2
Southern Latin America	1·3	0·9	1·6	6·9	5·7	8·1	3·1	1·9	4·8	16·9	11·9	23·5	3·8	2·6	5·5	21·1	16·0	27·0
Western Europe	9·4	7·0	12·4	7·5	6·3	8·6	25·6	15·3	41·9	20·2	13·8	29·0	28·5	18·4	42·6	22·5	16·6	29·5
High-income North America	8·0	6·0	10·4	6·6	5·6	7·6	27·1	18·1	40·7	22·4	17·0	29·7	29·0	19·5	41·7	23·9	18·3	30·4
Australasia	0·7	0·5	0·9	9·5	8·1	10·8	1·7	1·1	2·6	22·9	17·0	30·5	1·9	1·3	2·7	25·2	19·7	31·4
High-income Asia Pacific	2·7	2·0	3·5	5·4	4·5	6·1	9·8	5·7	16·3	19·6	13·1	28·4	9·1	5·8	13·8	18·1	13·2	24·0

*Estimates by income classification may not sum to the estimates at the global level, as not all economies are classified by income level by the World Bank. DALYs: disability-adjusted life years. GBD: Global Burden of Disease.

Under the three approaches, we calculated the economic value of mental disorder losses (Table 3). Using GDP per capita as a proxy for the value per DALY, economic losses due to mental disorders were estimated at 4·7 trillion USD using our composite approach. This estimate is 1·1 trillion USD larger than that reached using the 2016 reallocation approach and over 3·3 trillion USD larger than that reached from the unadjusted GBD 2019 estimates. Further adjusting for purchasing power parity, the global value of mental illness losses would exceed 7·2 trillion international dollars in 2019 (Table S7).

**Table 3:** Global economic value associated with premature mortality and morbidity from mental disorders, by estimation approach and value per DALY. Estimates are in trillions 2019 USD.

	Original approach			Reallocation approach			Composite approach		
Value per DALY (USD, 2019)	Estimate	Lower bound	Upper bound	Estimate	Lower bound	Upper bound	Estimate	Lower bound	Upper bound
1*x* GDP/capita	1·42	1·06	1·85	3·64	2·25	5·73	4·74	3·14	6·90
3*x* GDP/capita	4·27	3·17	5·55	10·93	6·76	17·20	14·22	9·42	20·71

DALY: disability-adjusted life year. USD: United States dollar. GDP: gross domestic product.

**Figure 2:**
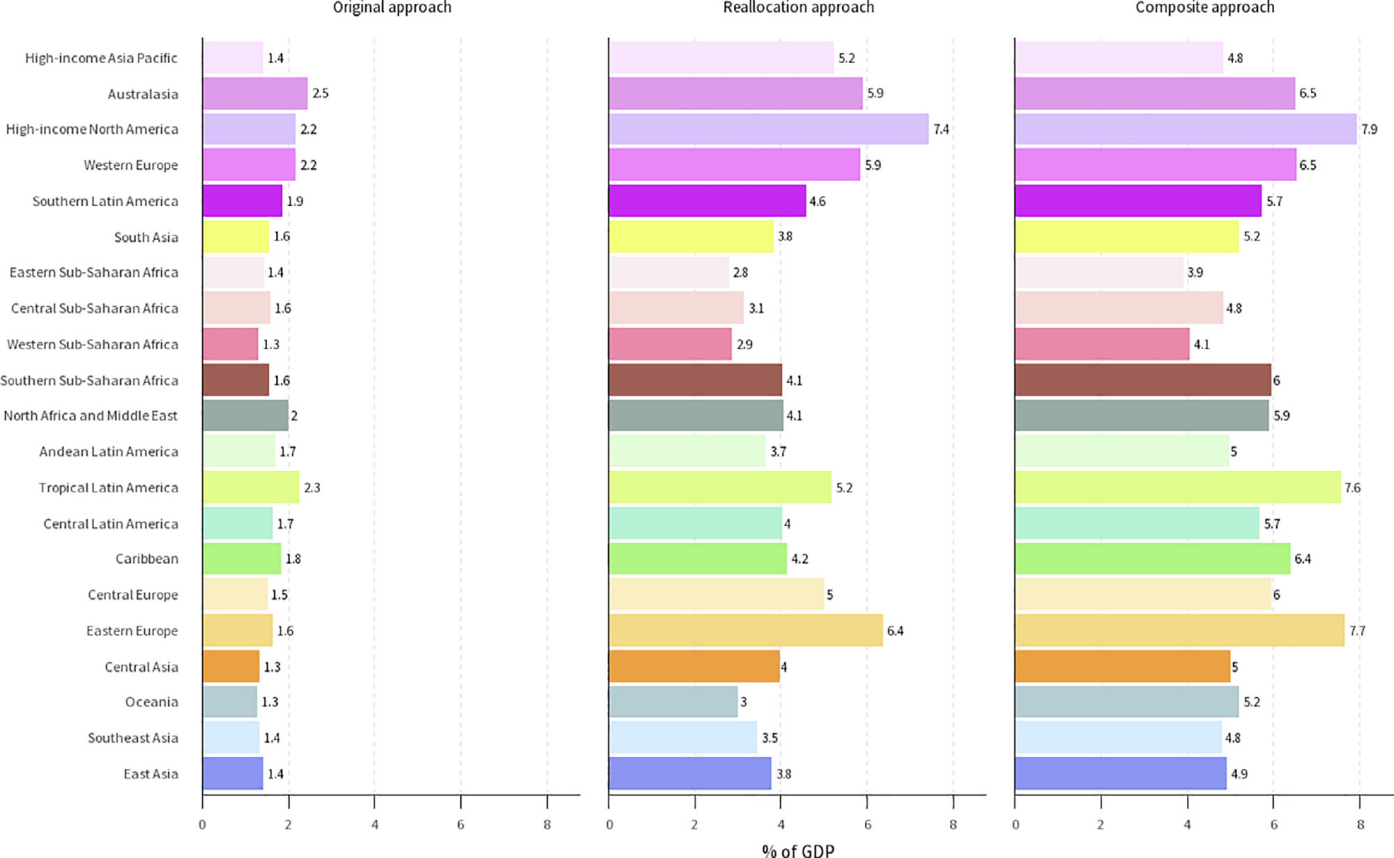
Economic burden of mental disorders, as a percent of GDP. The economic value is determined by using GDP per capita (USD 2019) as the value of a DALY. Values are aggregated by GBD region. GDP: gross domestic product; USD: United States dollar; DALY: disability-adjusted life year; GBD: Global Burden of Disease.

Although economic losses do not represent an actual loss to GDP, a sense of the scale can be gained by expressing the economic consequences with respect to GDP. Figure 2 displays the economic burden of disease due to mental disorders under the three estimation approaches by GBD region, as a percent of regional GDP. (Estimates by absolute values per DALY are provided in the Supplementary appendix, along with mapped data visualizing estimates across all values per DALY.) Across approaches, the greatest change in estimated burden occurs in Eastern Europe, Latin America, North America, and Southern sub-Saharan Africa. Under the relative GDP-per-capita values the economic burden would account for between 4% of GDP in Eastern Sub-Saharan Africa and 8% in High-income North America under our composite approach.

## Discussion

This study explores possible alternative approaches to estimating the global burden of mental illness and the economic losses thereof. In particular, we propose a composite approach to address contention in the classification of mental disorders. This approach suggests that the global DALYs attributable to mental disorders could exceed 400 million per year, or 16% of the total burden.

When applied against an economic value per DALY of one times GDP per capita, this approach further suggests that the per year losses associated with this burden could exceed 4·7 trillion USD in 2019. When adjusting for the uncertainty in estimates of the attributable burden of disease, the losses could range from 3·1 trillion to more than 6·9 trillion USD. Adjusting for purchasing power parity would increase the magnitude of these estimates, with ranges from 4·8 to 10·6 trillion international dollars at the global scale.

Put in context of the existing literature, our epidemiological and economic estimates provide two important contributions. First, our findings echo in magnitude those of Vigo and colleagues,^14^ which have highlighted that suicide and premature mortality due to mental disorders are potentially large sources of underestimation in the current GBD classification. Second, when including these sources of attributable mortality, the economic findings suggest staggering loses. We estimate that in 2019, the losses would already be over 1·8 trillion USD greater than Bloom and colleagues’ global losses projections for 2030 (2·9 trillion 2019 USD, using the same value per DALY approach).^12^

Our findings add to a growing literature concerning the classification of mental disorders, in particular related to underscoring the importance of including premature mortality attributable to mental disorders in burden conceptualizations.^14^^,^^18^^,^^31^^,^^32^ These calls have most recently been emphasized by GBD collaborators who have urged that “the differential mortality gap for individuals with mental disorders needs to be reflected within the GBD framework.”^16^ Our composite approach to assigning attributable mortality presents one potential attempt for acknowledging this differential mortality gap. Our economic analysis further provides updated monetary estimates of the burden of mental illness; to our knowledge, this is the first such analysis of the global economic burden of mental disorders in over a decade.

Our results should, however, be interpreted with several limitations in mind. First, our estimation approaches themselves all draw upon modeled data (i.e., GBD estimates). While GBD generates descriptions of morbidity and mortality at fine demographic and geographic levels, it is important to emphasize that the sophisticated modeling approaches implemented often draw on (potentially little) available underlying empirical data.^33^ These inputs can be extremely limited for particular diseases and geographical locations, especially so for mental disorders. By way of example, the GBD 2019 Data Input Sources Tool retrieves 3,084 separate data sources for mental disorders. Of these, only 60 pertain to sub-Saharan Africa (1·9%) and 58 to South Asia (1·8%).^34^ By comparison, of the 6,064 records pertaining to maternal and neonatal disorders, 631 are for sub-Saharan Africa (10·4%) and 270 for South Asia (4·5%). These severely limited inputs reflect a dearth of global mental health data; as of 2017, the World Mental Health survey initiative had conducted interviews in just 26 countries, only 13 of which were classified as low- or middle-income.^35^

Relatedly, our composite approach relies on pooled estimates of the relative risk of mortality from a systematic review and meta-analysis that itself is limited by the available data it draws upon.^15^ The review identified 203 studies for inclusion, of which only two were located in Africa, 16 in Asia, and one in South America. While the authors found that the estimates of mortality risk did not vary by region, the limited representation of studies from the world's most populous and epidemiologically diverse continents is a considerable shortcoming. It is possible, for instance, that the relative risk of all-cause mortality associated with mental disorders is lower where the burden of mortality is more heavily concentrated among child, maternal, and infectious diseases, and is higher where the burden is dominated by NCDs. Therefore, to reach a conservative estimate of attributable mortality, we separately estimated population attributable fractions for natural and unnatural causes of death and restricted our allocation of YLLs from natural causes to NCDs—meaning no deaths from maternal or infectious diseases were attributed to mental disorders under the composite approach.

Furthermore, our composite approach allocates mortality due to mental disorders by calculating population attributable fractions using the conventional formula, which may be biased in the presence of confounding or effect heterogeneity.^36^ In particular, the use of adjusted risk ratios (as in the current analysis) may result in anticonservative bias if the crude risk ratios are lower than the adjusted ones. To mitigate the potential for bias, our sensitivity analysis presents results under conservative assumptions for risk ratios and estimates of prevalence and mortality.

Despite these limitations, our findings underscore both that the true burden of mental disorders may only partially be captured by current estimation approaches, and that, consequently, the associated economic losses may be much higher than previously estimated. We note that our findings may themselves be an underestimate, as our composite approach excludes deaths due to neonatal, maternal, and infectious diseases attributable to mental disorders. However, we observe that conventional estimation approaches may fail to capture large shares of premature mortality attributable to mental health causes, both from self-inflicted and unnatural causes of death and mortality from NCDs. Capturing this share of the burden emphasizes that mental health is a critical risk factor for premature mortality, as well as a direct source of morbidity.

The magnitude of economic costs associated with mental disorders raises the need for health economics research, particularly on returns on investment and costing for effective prevention and treatment strategies.^37^ Further work is also needed to strengthen the measurement of the global burden of mental illness, not only for more fully capturing the morbidity and mortality of mental disorders, but also for incorporating the impacts of new and evolving threats—such as pandemics, conflicts, and climate change—to population mental health.

Our study emphasizes that mental health—far from being an issue solely concentrated in high-income regions alone— is a major global issue, one that imposes a significant toll to health and welfare. The large magnitude of these twin burdens highlights the urgency for global action to support mental health financing and to bolster its prioritization.

## Contributors

All authors contributed to study conception, methodology, and interpretation. DA oversaw data acquisition, programming, formal analysis, visualization, the first draft of the manuscript. All authors contributed to critical revision of the manuscript, with responses to reviewers and subsequent revisions led by DA. All authors had access to and verified all the data and accept responsibility for the decision to submit for publication.

## Data sharing statement

GBD estimates are available for download from the Global Health Data Exchange and are available freely for non-commercial users under the Open Data Commons Attribution License (https://ghdx.healthdata.org/gbd-2019). All codes used for the analysis in this article are available on GitHub (https://github.com/darias5/gmh_econ).

## Declaration of interests

We declare no competing interests.

## Appendix Supplementary materials

Image, application 1

## References

[1] World Health Organization (2013). https://apps.who.int/iris/bitstream/handle/10665/89966/9789241506021_eng.pdf?sequence=1.

[2] Rehm J, Shield KD (2019). Global burden of disease and the impact of mental and addictive disorders. Curr Psychiatry Rep.

[3] Charlson FJ, Baxter AJ, Dua T, Degenhardt L, Whiteford HA, Vos T. (2015). Excess mortality from mental, neurological and substance use disorders in the global burden of disease study 2010. Epidemiol Psychiatr Sci.

[4] Patel V, Saxena S, Lund C (2018). The Lancet commission on global mental health and sustainable development. Lancet.

[5] Vindegaard N, Benros ME. (2020). COVID-19 pandemic and mental health consequences: systematic review of the current evidence. Brain, Behav Immunity.

[6] Patel V, Boyce N, Collins PY, Saxena S, Horton R. (2011). A renewed agenda for global mental health. The Lancet.

[7] Trautmann S, Rehm J, Wittchen H-U. (2016). The economic costs of mental disorders: do our societies react appropriately to the burden of mental disorders?. EMBO Reports.

[8] Patel V, Kleinman A. (2003). Poverty and common mental disorders in developing countries. Bull World Health Organ.

[9] Lund C, Breen A, Flisher AJ (2010). Poverty and common mental disorders in low and middle income countries: A systematic review. Soc Sci Med.

[10] Canavan ME, Sipsma HL, Adhvaryu A (2013). Psychological distress in Ghana: associations with employment and lost productivity. Int J Mental Health Syst.

[11] Chisholm D, Sweeny K, Sheehan P (2016). Scaling-up treatment of depression and anxiety: a global return on investment analysis. Lancet Psychiatry.

[12] Bloom DE, Cafiero ET, Jané-Llopis E (2011). http://www3.weforum.org/docs/WEF_Harvard_HE_GlobalEconomicBurdenNonCommunicableDiseases_2011.pdf.

[13] The Lancet Global Health (2020). Mental health matters. Lancet Global Health.

[14] Vigo D, Thornicroft G, Atun R. (2016). Estimating the true global burden of mental illness. Lancet Psychiatry.

[15] Walker ER, McGee RE, Druss BG. (2015). Mortality in mental disorders and global disease burden implications: a systematic review and meta-analysis. JAMA Psychiatry.

[16] GBD 2019 Mental Disorders Collaborators (2022). Global, regional, and national burden of 12 mental disorders in 204 countries and territories, 1990-2019: a systematic analysis for the global burden of disease study 2019. Lancet Psychiatry.

[17] Vos T, Lim SS, Abbafati C (2020). Global burden of 369 diseases and injuries in 204 countries and territories, 1990–2019: a systematic analysis for the Global Burden of Disease Study 2019. Lancet.

[18] Whiteford HA, Ferrari AJ, Vos T. (2016). Challenges to estimating the true global burden of mental disorders. Lancet Psychiatry.

[19] Atun R, Vigo D, Thornicroft G. (2016). Challenges to estimating the true global burden of mental disorders – authors’ reply. Lancet Psychiatry.

[20] Bertolote JM, Fleischmann A. (2002). Suicide and psychiatric diagnosis: a worldwide perspective. World Psychiatry.

[21] Crump C, Sundquist K, Winkleby MA, Sundquist J. (2013). Mental disorders and risk of accidental death. Br J Psychiatry.

[22] Colton CW, Manderscheid RW. (2006). Congruencies in increased mortality rates, years of potential life lost, and causes of death among public mental health clients in eight states. Prev Chronic Dis.

[23] Robinson LA, Hammitt JK, O'Keeffe L (2019). Valuing mortality risk reductions in global benefit-cost analysis. J Benefit-Cost Anal.

[24] Jamison DT, Summers LH, Alleyne G (2013). Global health 2035: a world converging within a generation. Lancet.

[25] Khadka A, Verguet S. (2021). The economic value of changing mortality risk in low- and middle-income countries: a systematic breakdown by cause of death. BMC Med.

[26] Copenhagen Consensus Center. Methodology. https://www.copenhagenconsensus.com/scorecard-humanity/methodology Accessed 7 February 2021.

[27] Commission on Macroeconomics and Health (2001).

[28] Bertram MY, Lauer JA, De Joncheere K (2016). Cost–effectiveness thresholds: pros and cons. Bull World Health Organ.

[29] Jamison DT, Jha P, Laxminarayan R, Ord T., Lomborg B (2013). Global Problems, Smart Solutions.

[30] Jha P, Hum R, Gauvreau CL, Jordan K., Lomborg B (2018). Prioritizing Development.

[31] Vigo D, Jones L, Thornicroft G, Atun R. (2020). Burden of mental, neurological, substance use disorders and self-harm in North America: a comparative epidemiology of Canada, Mexico, and the United States. Can J Psychiatry.

[32] Vigo D, Jones L, Atun R, Thornicroft G. (2022). The true global disease burden of mental illness: still elusive. Lancet Psychiatry.

[33] Tichenor M, Sridhar D. (2020). Metric partnerships: global burden of disease estimates within the World Bank, the World Health Organisation and the Institute for Health Metrics and Evaluation. Wellcome Open Res.

[34] Institute for Health Metrics and Evaluation (IHME). Global Burden of Disease Study 2019 (GBD 2019) Data Input Sources Tool. http://ghdx.healthdata.org/gbd-2019/data-input-sources Accessed 27 April 2021.

[35] Stein DJ, Lim CCW, Roest AM (2017). The cross-national epidemiology of social anxiety disorder: data from the world mental health survey initiative. BMC Med.

[36] Darrow LA, Steenland NK. (2011). Confounding and bias in the attributable fraction. Epidemiology.

[37] Patel V, Chisholm D, Parikh R (2016). Addressing the burden of mental, neurological, and substance use disorders: key messages from disease control priorities, 3rd edition. Lancet.

