# Supplementary material for: Quantifying the global burden of mental disorders and their economic value
Source: eClinicalMedicine. 2022 Sep 28;54:101675. doi: 10.1016/j.eclinm.2022.101675 (PMC9526145; doi:10.1016/j.eclinm.2022.101675)
Supplement: Supplementary file 1 [file mmc1.pdf]

Supplementary appendix

Table S1. The global economic burden of mental disorders, by year and approach, calculated by Bloom and colleagues.[1] and adjusted to 2019 USD using gross domestic product deflator adjustment. Estimates are in trillions.

Year	Cost of illness (COI)			Value of lost output (VLO)	Value of losses using VSL approach †	
	Direct costs	Indirect costs	Total		1 DALY = 1*x* GDP per capita	1 DALY= 3*x* GDP per capita
2010	0·96	1·96	2·92	19·09*	1·57	4·72
2030	2·34	4·74	7·08		2·92	8·75
Average	1·50	3·05	4·55		2·14	6·42

* Annual VLO estimates are not reported by disease category. The estimate of VLO is the total for the range 2010-2030 for mental disorders.
† Authors' calculations based on Bloom and colleagues.[1]

DALY: disability-adjusted life year. GDP: gross domestic product. VSL: value of a statistical life.

Table S2. The economic value of mental disorder losses, using VSL approach, by year and monetary value assigned to one DALY. Authors' calculations based on Bloom and colleagues.[1] and adjusted to 2019 USD using gross domestic product deflator adjustment. Estimates are in trillions.

World Bank income group	1 DALY= 1x GDP per capita		1 DALY= 3x GDP per capita	
	2010	2030	2010	2030
High-income	1·02	1·32	3·05	3·97
Upper-middle-income	0·35	1·18	1·05	3·53
Lower-middle-income	0·17	0·36	0·50	1·09
Low-income	0·04	0·07	0·11	0·22
Global	1·57	2·92	4·72	8·75

DALY: disability-adjusted life year. VSL: value of a statistical life. GDP: gross domestic product.

Table S3. Hierarchical classification of mental disorders in the Global Burden of Disease (GBD) 2019 study.[2]

Level 1	Level 2	Level 3	Level 4
Non-communicable diseases			
	Mental disorders		
		Schizophrenia	
		Depressive disorders	
			Major depressive disorder
			Dysthymia
		Bipolar disorder	
		Anxiety disorders	
		Eating disorders	
			Anorexia nervosa
			Bulimia nervosa
		Autism spectrum disorders	
		Attention-deficit/hyperactivity disorder	
		Conduct disorder	
		Idiopathic developmental intellectual disability	
		Other mental disorders	
Not classified as “mental disorders” under GBD 2019			
	Substance use disorders		
		Alcohol use disorders	
		Drug use disorders	
			Opioid use disorders
			Cocaine use disorders
			Amphetamine use disorders
			Cannabis use disorders
			Other drug use disorders
	Neurological disorders		
		Alzheimer's disease and other dementias	
		Parkinson's disease	
		Idiopathic epilepsy	
		Multiple sclerosis	
		Motor neuron disease	
		Headache disorders	
			Migraine
			Tension-type headache
		Other neurological disorders	
	Musculoskeletal disorders		
Injuries			
	Self-harm and interpersonal violence		
		Self-harm	
			Self-harm by firearm
			Self-harm by other specified means

Table S4. Years lived with disability (YLDs) attributable to mental disorders as totals (millions) and percentages of overall burden, by World Bank income group classification and GBD region, under three estimation approaches.

	Original approach						Reallocation approach						Composite approach					
	YLDs			% of burden			YLDs			% of burden			YLDs			% of burden		
	Estimate	Lower bound	Upper bound	Estimate	Lower bound	Upper bound	Estimate	Lower bound	Upper bound	Estimate	Lower bound	Upper bound	Estimate	Lower bound	Upper bound	Estimate	Lower bound	Upper bound
Global	125·3	93·0	163·2	14·6	14·5	14·7	247·2	140·2	401·0	28·7	21·9	36·2	247·2	140·2	401·0	28·7	21·9	36·2
High income	24·5	18·1	32·0	14·1	14·0	14·3	53·9	30·7	87·5	31·1	23·7	39·2	53·9	30·7	87·5	31·1	23·7	39·2
Upper-middle income	45·6	33·8	59·7	14·5	14·4	14·6	92·8	51·9	152·6	29·5	22·1	37·4	92·8	51·9	152·6	29·5	22·1	37·4
Lower-middle income	44·9	33·0	58·9	14·5	14·3	14·6	83·5	45·6	138·7	26·9	19·8	34·4	83·5	45·6	138·7	26·9	19·8	34·4
Low income	10·1	7·4	13·5	16·5	16·3	16·7	16·8	9·4	27·5	27·3	20·8	34·1	16·8	9·4	27·5	27·3	20·8	34·1
East Asia	21·0	15·7	27·3	13·2	13·1	13·2	44·1	24·8	72·2	27·7	20·8	35·0	44·1	24·8	72·2	27·7	20·8	35·0
Southeast Asia	9·1	6·7	11·9	13·4	13·5	13·6	19·5	9·9	34·2	28·9	19·9	39·0	19·5	9·9	34·2	28·9	19·9	39·0
Oceania	0·2	0·1	0·2	13·6	13·6	13·7	0·3	0·2	0·5	26·0	18·6	34·0	0·3	0·2	0·5	26·0	18·6	34·0
Central Asia	1·3	0·9	1·7	14·2	14·2	14·4	2·7	1·5	4·5	30·2	22·4	38·7	2·7	1·5	4·5	30·2	22·4	38·7
Eastern Europe	3·5	2·6	4·5	12·7	12·7	12·9	8·0	4·7	12·9	29·4	23·2	36·6	8·0	4·7	12·9	29·4	23·2	36·6
Central Europe	1·7	1·3	2·3	11·6	11·5	11·7	4·0	2·3	6·6	26·8	20·2	34·2	4·0	2·3	6·6	26·8	20·2	34·2
Caribbean	0·8	0·6	1·0	16·7	16·4	16·9	1·4	0·8	2·2	29·2	22·2	36·6	1·4	0·8	2·2	29·2	22·2	36·6
Central Latin America	4·1	3·0	5·4	15·8	15·5	16·0	8·0	4·4	13·2	30·6	22·8	38·9	8·0	4·4	13·2	30·6	22·8	38·9
Tropical Latin America	5·1	3·7	6·6	18·8	18·7	19·0	9·6	5·5	15·5	35·6	27·4	44·6	9·6	5·5	15·5	35·6	27·4	44·6
Andean Latin America	1·1	0·8	1·4	17·5	17·1	17·9	1·9	1·1	3·1	30·6	23·6	38·0	1·9	1·1	3·1	30·6	23·6	38·0
North Africa and Middle East	10·7	7·8	14·1	19·4	19·2	19·8	18·7	10·4	30·7	34·0	25·6	43·0	18·7	10·4	30·7	34·0	25·6	43·0
Southern Sub-Saharan Africa	1·2	0·9	1·6	14·1	13·9	14·5	2·4	1·4	3·8	27·1	20·9	33·6	2·4	1·4	3·8	27·1	20·9	33·6
Western Sub-Saharan Africa	6·7	4·9	9·0	14·5	14·4	14·7	12·2	6·5	20·7	26·3	19·0	34·0	12·2	6·5	20·7	26·3	19·0	34·0
Central Sub-Saharan Africa	2·1	1·5	2·8	16·8	16·5	17·1	3·4	1·9	5·6	27·4	20·7	34·3	3·4	1·9	5·6	27·4	20·7	34·3
Eastern Sub-Saharan Africa	5·8	4·2	7·7	16·5	16·2	16·8	9·4	5·5	15·0	26·6	21·1	32·5	9·4	5·5	15·0	26·6	21·1	32·5
South Asia	28·8	21·2	37·6	14·1	14·0	14·2	52·7	29·1	86·8	25·9	19·1	32·8	52·7	29·1	86·8	25·9	19·1	32·8
Southern Latin America	1·3	0·9	1·6	16·5	16·3	16·7	2·4	1·4	3·8	31·2	24·3	38·6	2·4	1·4	3·8	31·2	24·3	38·6
Western Europe	9·4	7·0	12·4	15·4	15·3	15·6	19·4	10·9	31·9	31·7	24·0	40·4	19·4	10·9	31·9	31·7	24·0	40·4
High-income North America	8·0	6·0	10·4	13·7	13·6	13·9	19·2	11·2	30·5	33·0	25·6	41·0	19·2	11·2	30·5	33·0	25·6	41·0
Australasia	0·7	0·5	0·9	17·7	17·5	18·0	1·3	0·8	2·0	32·6	26·2	39·3	1·3	0·8	2·0	32·6	26·2	39·3
High-income Asia Pacific	2·7	2·0	3·5	10·9	10·8	11·0	6·4	3·6	10·5	25·8	19·6	33·0	6·4	3·6	10·5	25·8	19·6	33·0

* Estimates by income classification may not sum to the estimates at the global level, as not all economies are classified by income level by the World Bank. YLDs: years lived with disability. GBD: Global Burden of Disease.

Table S5. Years of life lost (YLLs) attributable to mental disorders as totals (millions) and percentages of overall burden, by World Bank income group classification and GBD region, under three estimation approaches.

	Original approach						Reallocation approach						Composite approach					
	YLLs			% of burden			YLLs			% of burden			YLLs			% of burden		
	Estimate	Lower bound	Upper bound	Estimate	Lower bound	Upper bound	Estimate	Lower bound	Upper bound	Estimate	Lower bound	Upper bound	Estimate	Lower bound	Upper bound	Estimate	Lower bound	Upper bound
Global	-	-	-	-	-	-	74·0	55·0	108·6	4·4	3·5	6·0	170·5	136·5	207·3	10·2	8·7	11·4
High income	-	-	-	-	-	-	20·4	14·3	31·9	10·6	7·7	15·7	27·1	22·0	32·8	14·0	11·9	16·1
Upper-middle income	-	-	-	-	-	-	24·7	15·9	41·5	4·9	3·6	7·2	63·8	47·4	83·5	12·7	10·8	14·5
Lower-middle income	-	-	-	-	-	-	25·3	17·8	37·1	3·4	2·8	4·4	63·9	44·6	86·9	8·7	6·9	10·2
Low income	-	-	-	-	-	-	3·5	2·2	5·6	1·5	1·1	1·9	15·5	10·4	22·5	6·5	5·2	7·8
East Asia	-	-	-	-	-	-	11·7	7·0	20·9	4·9	3·4	7·7	28·5	20·8	37·6	12·0	10·1	13·8
Southeast Asia	-	-	-	-	-	-	3·8	2·2	6·5	2·9	2·0	4·3	12·8	8·8	17·8	9·8	8·0	11·7
Oceania	-	-	-	-	-	-	0·1	0·0	0·1	1·7	1·2	2·2	0·3	0·2	0·5	8·2	6·6	9·9
Central Asia	-	-	-	-	-	-	1·1	0·8	1·5	5·4	4·6	6·4	2·0	1·5	2·7	10·2	8·6	11·9
Eastern Europe	-	-	-	-	-	-	5·4	4·0	7·8	8·8	7·4	11·1	8·1	6·0	10·6	13·1	11·0	15·1
Central Europe	-	-	-	-	-	-	1·7	1·1	2·9	6·7	5·0	9·6	2·8	1·9	3·9	10·9	9·2	12·7
Caribbean	-	-	-	-	-	-	0·4	0·2	0·6	3·8	2·8	5·2	1·3	0·9	1·9	13·0	10·8	15·2
Central Latin America	-	-	-	-	-	-	2·1	1·3	3·4	5·1	4·0	6·9	6·2	4·4	8·6	15·3	13·1	17·6
Tropical Latin America	-	-	-	-	-	-	2·0	1·4	3·2	5·0	3·7	7·6	7·4	6·2	8·6	18·3	16·1	20·5
Andean Latin America	-	-	-	-	-	-	0·4	0·2	0·7	4·6	3·2	6·3	1·3	0·8	1·9	13·6	11·4	16·0
North Africa and Middle East	-	-	-	-	-	-	2·8	1·7	4·9	3·5	2·5	5·2	12·6	8·9	17·4	15·9	13·5	18·4
Southern Sub-Saharan Africa	-	-	-	-	-	-	0·8	0·6	1·2	2·8	2·2	3·6	2·3	1·7	3·1	7·9	6·5	9·3
Western Sub-Saharan Africa	-	-	-	-	-	-	2·5	1·5	4·1	1·1	0·8	1·6	8·6	5·4	12·8	4·0	3·0	5·0
Central Sub-Saharan Africa	-	-	-	-	-	-	0·7	0·5	1·1	1·6	1·2	2·0	2·9	1·9	4·3	6·5	5·0	8·0
Eastern Sub-Saharan Africa	-	-	-	-	-	-	1·9	1·3	3·0	1·5	1·2	1·9	6·4	4·4	9·2	4·9	3·9	5·9
South Asia	-	-	-	-	-	-	18·0	13·3	25·3	4·2	3·5	5·3	43·3	30·8	57·4	10·1	8·1	12·0
Southern Latin America	-	-	-	-	-	-	0·7	0·5	1·0	6·5	5·0	9·5	1·5	1·2	1·7	13·8	11·9	15·6
Western Europe	-	-	-	-	-	-	6·1	4·0	10·4	9·4	6·1	15·6	9·1	7·5	10·7	13·8	11·7	16·0
High-income North America	-	-	-	-	-	-	7·9	6·5	10·6	12·6	10·5	16·8	9·8	8·3	11·2	15·5	13·3	17·7
Australasia	-	-	-	-	-	-	0·4	0·3	0·6	11·7	8·5	17·7	0·6	0·5	0·7	16·8	14·4	19·2
High-income Asia Pacific	-	-	-	-	-	-	3·4	1·9	6·0	13·5	7·7	23·2	2·7	2·2	3·2	10·6	8·7	12·5

* Estimates by income classification may not sum to the estimates at the global level, as not all economies are classified by income level by the World Bank. YLLs: years of life lost. GBD: Global Burden of Disease.

Table S6. Deaths attributable to mental disorders as totals (millions) and percentages of overall burden, by World Bank income classification and GBD region, under three estimation approaches.

	Original approach						Reallocation approach						Composite approach					
	Deaths			% of burden			Deaths			% of burden			Deaths			% of burden		
	Estimate	Lower bound	Upper bound	Estimate	Lower bound	Upper bound	Estimate	Lower bound	Upper bound	Estimate	Lower bound	Upper bound	Estimate	Lower bound	Upper bound	Estimate	Lower bound	Upper bound
Global	-	-	-	-	-	-	3·2	1·8	5·9	5·6	3·3	9·9	5·9	4·7	7·1	10·4	8·8	12·0
High income	-	-	-	-	-	-	1·1	0·6	2·2	10·2	5·3	19·1	1·4	1·1	1·7	12·3	10·3	14·3
Upper-middle income	-	-	-	-	-	-	1·1	0·6	2·3	5·4	3·1	9·6	2·3	1·7	3·0	11·1	9·3	12·8
Lower-middle income	-	-	-	-	-	-	0·8	0·5	1·4	3·9	2·7	6·2	1·9	1·3	2·6	9·6	7·7	11·4
Low income	-	-	-	-	-	-	0·1	0·1	0·2	2·4	1·5	3·8	0·4	0·2	0·5	7·7	6·2	9·3
East Asia	-	-	-	-	-	-	0·6	0·3	1·2	5·3	3·0	9·4	1·2	0·9	1·5	10·5	8·8	12·2
Southeast Asia	-	-	-	-	-	-	0·2	0·1	0·3	3·8	2·0	6·8	0·4	0·3	0·6	9·2	7·5	11·0
Oceania	-	-	-	-	-	-	0·0	0·0	0·0	2·1	1·4	3·1	0·0	0·0	0·0	8·7	7·1	10·4
Central Asia	-	-	-	-	-	-	0·0	0·0	0·1	4·7	3·4	6·9	0·1	0·0	0·1	9·6	8·0	11·2
Eastern Europe	-	-	-	-	-	-	0·2	0·1	0·3	7·1	4·9	11·2	0·3	0·2	0·4	11·2	9·4	13·0
Central Europe	-	-	-	-	-	-	0·1	0·0	0·2	6·6	3·7	11·4	0·1	0·1	0·2	9·9	8·2	11·5
Caribbean	-	-	-	-	-	-	0·0	0·0	0·0	5·2	3·1	8·4	0·0	0·0	0·1	12·7	10·6	15·0
Central Latin America	-	-	-	-	-	-	0·1	0·0	0·2	6·4	3·6	10·8	0·2	0·1	0·2	12·6	10·6	14·8
Tropical Latin America	-	-	-	-	-	-	0·1	0·0	0·2	6·5	3·5	12·5	0·2	0·2	0·3	15·5	13·4	17·7
Andean Latin America	-	-	-	-	-	-	0·0	0·0	0·0	6·1	3·4	10·3	0·0	0·0	0·1	12·1	10·0	14·3
North Africa and Middle East	-	-	-	-	-	-	0·1	0·1	0·3	4·4	2·4	8·0	0·4	0·3	0·5	14·3	11·9	16·7
Southern Sub-Saharan Africa	-	-	-	-	-	-	0·0	0·0	0·0	3·3	2·1	5·3	0·1	0·0	0·1	8·0	6·6	9·5
Western Sub-Saharan Africa	-	-	-	-	-	-	0·1	0·0	0·2	2·2	1·3	3·5	0·2	0·1	0·3	5·1	3·9	6·3
Central Sub-Saharan Africa	-	-	-	-	-	-	0·0	0·0	0·0	2·4	1·6	3·5	0·1	0·0	0·1	7·4	5·8	9·1
Eastern Sub-Saharan Africa	-	-	-	-	-	-	0·1	0·0	0·1	2·4	1·6	3·8	0·2	0·1	0·2	6·0	4·8	7·2
South Asia	-	-	-	-	-	-	0·5	0·3	0·9	4·3	3·0	6·5	1·3	0·9	1·7	10·6	8·4	12·5
Southern Latin America	-	-	-	-	-	-	0·0	0·0	0·1	6·7	3·7	12·8	0·1	0·0	0·1	11·6	9·8	13·3
Western Europe	-	-	-	-	-	-	0·4	0·2	0·8	9·5	4·6	19·2	0·5	0·4	0·6	12·7	10·6	14·8
High-income North America	-	-	-	-	-	-	0·3	0·2	0·6	10·4	6·4	17·8	0·4	0·4	0·5	13·7	11·7	15·8
Australasia	-	-	-	-	-	-	0·0	0·0	0·0	10·6	5·5	20·6	0·0	0·0	0·0	14·9	12·6	17·3
High-income Asia Pacific	-	-	-	-	-	-	0·2	0·1	0·5	14·3	6·1	29·2	0·2	0·1	0·2	9·0	7·3	10·7

* Estimates by income classification may not sum to the estimates at the global level, as not all economies are classified by income level by the World Bank. GBD: Global Burden of Disease.

Table S7. The global economic burden associated with premature mortality and morbidity from mental disorders, by estimation approach and value per DALY, using alternative valuations. Dollar amounts are in trillions.

Panel A: Using values per DALY of $1,000 and $5,000 (USD 2019)

	Original approach			Reallocation approach			Composite approach		
Value per DALY (USD, 2019)	Estimate	Lower bound	Upper bound	Estimate	Lower bound	Upper bound	Estimate	Lower bound	Upper bound
$1,000	0·13	0·09	0·16	0·32	0·20	0·51	0·42	0·28	0·61
$5,000	0·63	0·46	0·82	1·61	0·99	2·53	2·09	1·38	3·04

Panel B: Using GDP per capita, PPP-adjusted, per DALY (international dollars, 2019)

	Original approach			Reallocation approach			Composite approach		
Value per DALY (International dollar, 2019)	Estimate	Lower bound	Upper bound	Estimate	Lower bound	Upper bound	Estimate	Lower bound	Upper bound
1*x* GDP PPP/capita	2·18	1·62	2·84	5·60	3·46	8·80	7·28	4·82	10·60
3*x* GDP PPP/capita	6·55	4·86	8·53	16·79	10·38	26·41	21·84	14·47	31·80

DALY: disability-adjusted life year. USD: United States dollar. GDP: gross domestic product. PPP: purchasing power parity.

Table S8. The economic burden associated with premature mortality and morbidity from mental disorders, by estimation approach and value per DALY, using alternative valuations and by World Bank income classification and GBD region. Dollar amounts are in trillions.

Panel A: Using $1,000 per DALY (USD 2019)	Original approach			2016 reallocation approach			Composite approach		
	Estimate	Lower bound	Upper bound	Estimate	Lower bound	Upper bound	Estimate	Lower bound	Upper bound
Global	0·13	0·09	0·16	0·32	0·20	0·51	0·42	0·28	0·61
High income	0·02	0·02	0·03	0·07	0·05	0·12	0·08	0·05	0·12
Upper-middle income	0·05	0·03	0·06	0·12	0·07	0·19	0·16	0·10	0·24
Lower-middle income	0·04	0·03	0·06	0·11	0·06	0·17	0·15	0·09	0·23
Low income	0·01	0·01	0·01	0·02	0·01	0·03	0·03	0·02	0·05
East Asia	0·02	0·02	0·03	0·06	0·03	0·09	0·07	0·05	0·11
Southeast Asia	0·01	0·01	0·01	0·02	0·01	0·04	0·03	0·02	0·05
Oceania	-	-	-	-	-	-	-	-	-
Central Asia	-	-	-	-	-	0·01	-	-	0·01
Eastern Europe	-	-	-	0·01	0·01	0·02	0·02	0·01	0·02
Central Europe	-	-	-	0·01	-	0·01	0·01	-	0·01
Caribbean	-	-	-	-	-	-	-	-	-
Central Latin America	-	-	0·01	0·01	0·01	0·02	0·01	0·01	0·02
Tropical Latin America	0·01	-	0·01	0·01	0·01	0·02	0·02	0·01	0·02
Andean Latin America	-	-	-	-	-	-	-	-	-
North Africa and Middle East	0·01	0·01	0·01	0·02	0·01	0·04	0·03	0·02	0·05
Southern Sub-Saharan Africa	-	-	-	-	-	-	-	-	0·01
Western Sub-Saharan Africa	0·01	-	0·01	0·01	0·01	0·02	0·02	0·01	0·03
Central Sub-Saharan Africa	-	-	-	-	-	0·01	0·01	-	0·01

Eastern Sub-Saharan Africa	0·01	-	0·01	0·01	0·01	0·02	0·02	0·01	0·02
South Asia	0·03	0·02	0·04	0·07	0·04	0·11	0·10	0·06	0·14
Southern Latin America	-	-	-	-	-	-	-	-	0·01
Western Europe	0·01	0·01	0·01	0·03	0·02	0·04	0·03	0·02	0·04
High-income North America	0·01	0·01	0·01	0·03	0·02	0·04	0·03	0·02	0·04
Australasia	-	-	-	-	-	-	-	-	-
High-income Asia Pacific	-	-	-	0·01	0·01	0·02	0·01	0·01	0·01

Panel B: Using $5,000 per DALY (USD 2019)

	Original approach			2016 reallocation approach			Composite approach		
	Estimate	Lower bound	Upper bound	Estimate	Lower bound	Upper bound	Estimate	Lower bound	Upper bound
Global	0·63	0·46	0·82	1·61	0·99	2·53	2·09	1·38	3·04
High income	0·12	0·09	0·16	0·37	0·23	0·59	0·40	0·26	0·60
Upper-middle income	0·23	0·17	0·30	0·59	0·35	0·96	0·78	0·50	1·18
Lower-middle income	0·22	0·17	0·29	0·54	0·32	0·87	0·74	0·45	1·13
Low income	0·05	0·04	0·07	0·10	0·06	0·16	0·16	0·10	0·25
East Asia	0·10	0·08	0·14	0·28	0·16	0·46	0·36	0·23	0·55
Southeast Asia	0·05	0·03	0·06	0·12	0·06	0·20	0·16	0·09	0·26
Oceania	-	-	-	-	-	-	-	-	0·01
Central Asia	0·01	-	0·01	0·02	0·01	0·03	0·02	0·01	0·04
Eastern Europe	0·02	0·01	0·02	0·07	0·05	0·10	0·08	0·05	0·12
Central Europe	0·01	0·01	0·01	0·03	0·02	0·05	0·03	0·02	0·05
Caribbean	-	-	0·01	0·01	0·01	0·01	0·01	0·01	0·02
Central Latin America	0·02	0·02	0·03	0·05	0·03	0·08	0·07	0·04	0·11
Tropical Latin America	0·03	0·02	0·03	0·06	0·03	0·09	0·08	0·06	0·12
Andean Latin America	0·01	-	0·01	0·01	0·01	0·02	0·02	0·01	0·02

North Africa and Middle East	0·05	0·04	0·07	0·11	0·06	0·18	0·16	0·10	0·24
Southern Sub-Saharan Africa	0·01	-	0·01	0·02	0·01	0·02	0·02	0·02	0·03
Western Sub-Saharan Africa	0·03	0·02	0·04	0·07	0·04	0·12	0·10	0·06	0·17
Central Sub-Saharan Africa	0·01	0·01	0·01	0·02	0·01	0·03	0·03	0·02	0·05
Eastern Sub-Saharan Africa	0·03	0·02	0·04	0·06	0·03	0·09	0·08	0·05	0·12
South Asia	0·14	0·11	0·19	0·35	0·22	0·56	0·48	0·30	0·72
Southern Latin America	0·01	-	0·01	0·02	0·01	0·02	0·02	0·01	0·03
Western Europe	0·05	0·03	0·06	0·13	0·08	0·21	0·14	0·09	0·21
High-income North America	0·04	0·03	0·05	0·14	0·09	0·20	0·14	0·10	0·21
Australasia	-	-	-	0·01	0·01	0·01	0·01	0·01	0·01
High-income Asia Pacific	0·01	0·01	0·02	0·05	0·03	0·08	0·05	0·03	0·07
Panel C: Using 1x GDP per capita, per DALY (USD 2019)									
	Original approach			2016 reallocation approach			Composite approach		
	Estimate	Lower bound	Upper bound	Estimate	Lower bound	Upper bound	Estimate	Lower bound	Upper bound
Global	1·42	1·06	1·85	3·64	2·25	5·73	4·74	3·14	6·90
High income	1·12	0·83	1·46	3·46	2·19	5·41	3·72	2·45	5·48
Upper-middle income	0·40	0·29	0·52	1·04	0·62	1·69	1·37	0·87	2·07
Lower-middle income	0·10	0·07	0·13	0·24	0·14	0·38	0·32	0·20	0·49
Low income	0·01	-	0·01	0·01	0·01	0·02	0·02	0·01	0·03
East Asia	0·20	0·15	0·26	0·54	0·32	0·89	0·71	0·44	1·07
Southeast Asia	0·04	0·03	0·05	0·10	0·05	0·17	0·14	0·08	0·22
Oceania	-	-	-	-	-	-	-	-	-
Central Asia	0·01	-	0·01	0·02	0·01	0·02	0·02	0·01	0·03
Eastern Europe	0·03	0·02	0·04	0·13	0·09	0·20	0·16	0·10	0·23
Central Europe	0·03	0·02	0·03	0·08	0·05	0·14	0·10	0·06	0·15

Caribbean	0·01	-	0·01	0·01	0·01	0·02	0·02	0·01	0·03
Central Latin America	0·03	0·02	0·04	0·08	0·04	0·12	0·11	0·07	0·16
Tropical Latin America	0·04	0·03	0·06	0·10	0·06	0·16	0·14	0·10	0·20
Andean Latin America	0·01	-	0·01	0·01	0·01	0·02	0·02	0·01	0·03
North Africa and Middle East	0·08	0·06	0·10	0·16	0·09	0·26	0·23	0·14	0·36
Southern Sub-Saharan Africa	0·01	-	0·01	0·02	0·01	0·03	0·02	0·02	0·04
Western Sub-Saharan Africa	0·01	0·01	0·02	0·03	0·01	0·04	0·04	0·02	0·06
Central Sub-Saharan Africa	-	-	-	0·01	-	0·01	0·01	0·01	0·01
Eastern Sub-Saharan Africa	0·01	-	0·01	0·01	0·01	0·02	0·02	0·01	0·02
South Asia	0·05	0·04	0·07	0·13	0·08	0·21	0·18	0·11	0·27
Southern Latin America	0·01	0·01	0·02	0·04	0·02	0·06	0·04	0·03	0·06
Western Europe	0·40	0·29	0·52	1·07	0·64	1·76	1·20	0·77	1·79
High-income North America	0·51	0·38	0·66	1·72	1·15	2·58	1·84	1·24	2·65
Australasia	0·04	0·03	0·05	0·09	0·06	0·15	0·10	0·07	0·15
High-income Asia Pacific	0·10	0·08	0·13	0·37	0·22	0·62	0·34	0·22	0·52

Panel D: Using 3x GDP per capita, per DALY (USD 2019)

	Original approach			2016 reallocation approach			Composite approach		
	Estimate	Lower bound	Upper bound	Estimate	Lower bound	Upper bound	Estimate	Lower bound	Upper bound
Global	4·27	3·17	5·55	10·93	6·76	17·20	14·22	9·42	20·71
High income	3·35	2·48	4·37	10·37	6·56	16·23	11·16	7·35	16·43
Upper-middle income	1·19	0·88	1·56	3·13	1·86	5·08	4·12	2·62	6·20
Lower-middle income	0·29	0·21	0·38	0·71	0·42	1·14	0·96	0·59	1·48
Low income	0·02	0·01	0·02	0·04	0·02	0·06	0·06	0·04	0·09
East Asia	0·61	0·46	0·79	1·63	0·96	2·67	2·12	1·33	3·20
Southeast Asia	0·12	0·09	0·15	0·30	0·16	0·52	0·42	0·24	0·67

	Original approach			2016 reallocation approach			Composite approach		
Oceania	-	-	-	-	-	0·01	0·01	-	0·01
Central Asia	0·02	0·01	0·02	0·05	0·03	0·07	0·06	0·04	0·09
Eastern Europe	0·10	0·07	0·13	0·39	0·26	0·59	0·47	0·31	0·69
Central Europe	0·08	0·06	0·10	0·25	0·15	0·41	0·30	0·18	0·46
Caribbean	0·02	0·01	0·02	0·04	0·02	0·06	0·05	0·03	0·08
Central Latin America	0·09	0·07	0·12	0·23	0·13	0·37	0·32	0·20	0·49
Tropical Latin America	0·13	0·09	0·17	0·29	0·18	0·47	0·43	0·29	0·61
Andean Latin America	0·02	0·01	0·03	0·04	0·02	0·07	0·06	0·03	0·09
North Africa and Middle East	0·24	0·17	0·31	0·48	0·27	0·78	0·69	0·43	1·07
Southern Sub-Saharan Africa	0·02	0·01	0·03	0·05	0·03	0·08	0·07	0·05	0·11
Western Sub-Saharan Africa	0·04	0·03	0·05	0·08	0·04	0·13	0·11	0·06	0·18
Central Sub-Saharan Africa	0·01	0·01	0·01	0·02	0·01	0·03	0·03	0·02	0·04
Eastern Sub-Saharan Africa	0·02	0·01	0·02	0·03	0·02	0·05	0·05	0·03	0·07
South Asia	0·16	0·12	0·21	0·40	0·25	0·63	0·55	0·34	0·82
Southern Latin America	0·04	0·03	0·06	0·11	0·07	0·17	0·13	0·09	0·19
Western Europe	1·19	0·88	1·56	3·22	1·92	5·28	3·59	2·32	5·37
High-income North America	1·52	1·14	1·97	5·17	3·44	7·75	5·52	3·72	7·95
Australasia	0·12	0·09	0·16	0·28	0·18	0·44	0·31	0·21	0·45
High-income Asia Pacific	0·31	0·23	0·40	1·12	0·65	1·86	1·03	0·66	1·57

Panel E: Using 3x GDP per capita, PPP-adjusted, per DALY (international dollars, 2019)

	Original approach			2016 reallocation approach			Composite approach		
	Estimate	Lower bound	Upper bound	Estimate	Lower bound	Upper bound	Estimate	Lower bound	Upper bound
Global	2·18	1·62	2·84	5·60	3·46	8·80	7·28	4·82	10·60
High income	1·27	0·94	1·66	3·88	2·44	6·11	4·22	2·76	6·23
Upper-middle income	0·78	0·58	1·02	2·05	1·23	3·33	2·70	1·71	4·06

Lower-middle income	0·31	0·23	0·40	0·75	0·45	1·20	1·02	0·63	1·56
Low income	0·02	0·01	0·02	0·04	0·02	0·06	0·06	0·03	0·09
East Asia	0·33	0·25	0·43	0·89	0·52	1·46	1·16	0·73	1·75
Southeast Asia	0·11	0·08	0·14	0·28	0·15	0·49	0·39	0·23	0·63
Oceania	-	-	-	-	-	-	-	-	-
Central Asia	0·02	0·01	0·02	0·05	0·03	0·08	0·06	0·04	0·09
Eastern Europe	0·09	0·07	0·12	0·35	0·23	0·52	0·41	0·27	0·61
Central Europe	0·05	0·04	0·07	0·18	0·11	0·29	0·21	0·13	0·32
Caribbean	0·01	-	0·01	0·02	0·01	0·02	0·02	0·01	0·04
Central Latin America	0·06	0·05	0·08	0·16	0·09	0·25	0·22	0·14	0·34
Tropical Latin America	0·08	0·06	0·10	0·17	0·10	0·28	0·25	0·17	0·36
Andean Latin America	0·01	0·01	0·02	0·03	0·02	0·04	0·04	0·02	0·06
North Africa and Middle East	0·19	0·14	0·25	0·38	0·22	0·63	0·55	0·34	0·85
Southern Sub-Saharan Africa	0·02	0·01	0·02	0·04	0·02	0·06	0·06	0·04	0·08
Western Sub-Saharan Africa	0·03	0·02	0·04	0·06	0·03	0·10	0·09	0·05	0·14
Central Sub-Saharan Africa	0·01	-	0·01	0·01	0·01	0·02	0·02	0·01	0·03
Eastern Sub-Saharan Africa	0·02	0·01	0·02	0·03	0·02	0·05	0·04	0·03	0·06
South Asia	0·18	0·13	0·24	0·45	0·27	0·70	0·61	0·38	0·91
Southern Latin America	0·03	0·02	0·04	0·07	0·05	0·12	0·09	0·06	0·13
Western Europe	0·47	0·35	0·62	1·28	0·76	2·09	1·42	0·92	2·13
High-income North America	0·51	0·38	0·66	1·73	1·15	2·60	1·85	1·25	2·67
Australasia	0·04	0·03	0·05	0·09	0·06	0·14	0·10	0·07	0·14
High-income Asia Pacific	0·12	0·09	0·15	0·43	0·25	0·71	0·40	0·25	0·60

Panel F: Using 3x GDP per capita, PPP-adjusted, per DALY (international dollars, 2019)

	Original approach			2016 reallocation approach			Composite approach		

	Estimate	Lower bound	Upper bound	Estimate	Lower bound	Upper bound	Estimate	Lower bound	Upper bound
Global	6·55	4·86	8·53	16·79	10·38	26·41	21·84	14·47	31·80
High income	3·81	2·83	4·98	11·65	7·32	18·32	12·66	8·29	18·70
Upper-middle income	2·33	1·73	3·05	6·16	3·68	9·99	8·09	5·14	12·18
Lower-middle income	0·92	0·68	1·21	2·26	1·35	3·61	3·07	1·88	4·69
Low income	0·05	0·04	0·07	0·11	0·06	0·17	0·17	0·10	0·26
East Asia	1·00	0·75	1·30	2·67	1·57	4·38	3·47	2·18	5·24
Southeast Asia	0·33	0·24	0·43	0·85	0·45	1·47	1·17	0·68	1·89
Oceania	-	-	-	0·01	-	0·01	0·01	0·01	0·01
Central Asia	0·05	0·04	0·07	0·15	0·09	0·23	0·18	0·11	0·28
Eastern Europe	0·27	0·20	0·35	1·04	0·70	1·56	1·24	0·82	1·82
Central Europe	0·16	0·12	0·21	0·53	0·32	0·87	0·63	0·39	0·97
Caribbean	0·02	0·01	0·03	0·05	0·03	0·07	0·07	0·04	0·11
Central Latin America	0·19	0·14	0·25	0·47	0·28	0·76	0·66	0·41	1·01
Tropical Latin America	0·23	0·17	0·30	0·52	0·31	0·83	0·76	0·52	1·08
Andean Latin America	0·04	0·03	0·05	0·08	0·05	0·13	0·11	0·07	0·17
North Africa and Middle East	0·57	0·42	0·75	1·14	0·65	1·88	1·66	1·03	2·56
Southern Sub-Saharan Africa	0·05	0·03	0·06	0·12	0·07	0·18	0·17	0·11	0·25
Western Sub-Saharan Africa	0·09	0·06	0·11	0·19	0·10	0·31	0·26	0·15	0·43
Central Sub-Saharan Africa	0·02	0·01	0·03	0·04	0·02	0·06	0·06	0·04	0·09
Eastern Sub-Saharan Africa	0·05	0·03	0·06	0·09	0·05	0·14	0·12	0·08	0·19
South Asia	0·55	0·40	0·71	1·34	0·82	2·10	1·82	1·13	2·73
Southern Latin America	0·09	0·07	0·12	0·22	0·14	0·35	0·28	0·19	0·40
Western Europe	1·42	1·04	1·86	3·83	2·29	6·27	4·27	2·76	6·39

High-income North America	1·53	1·14	1·99	5·20	3·46	7·80	5·55	3·74	8·00
Australasia	0·11	0·08	0·15	0·27	0·17	0·42	0·30	0·20	0·43
High-income Asia Pacific	0·35	0·26	0·46	1·29	0·76	2·14	1·20	0·76	1·81

DALY: disability-adjusted life year. GDP: gross domestic product. PPP: purchasing power parity. USD: United States dollar.

Figure S1. Economic burden of mental disorders, as a percent of GDP, by absolute and relative values per DALY. Values are aggregated by GBD region.

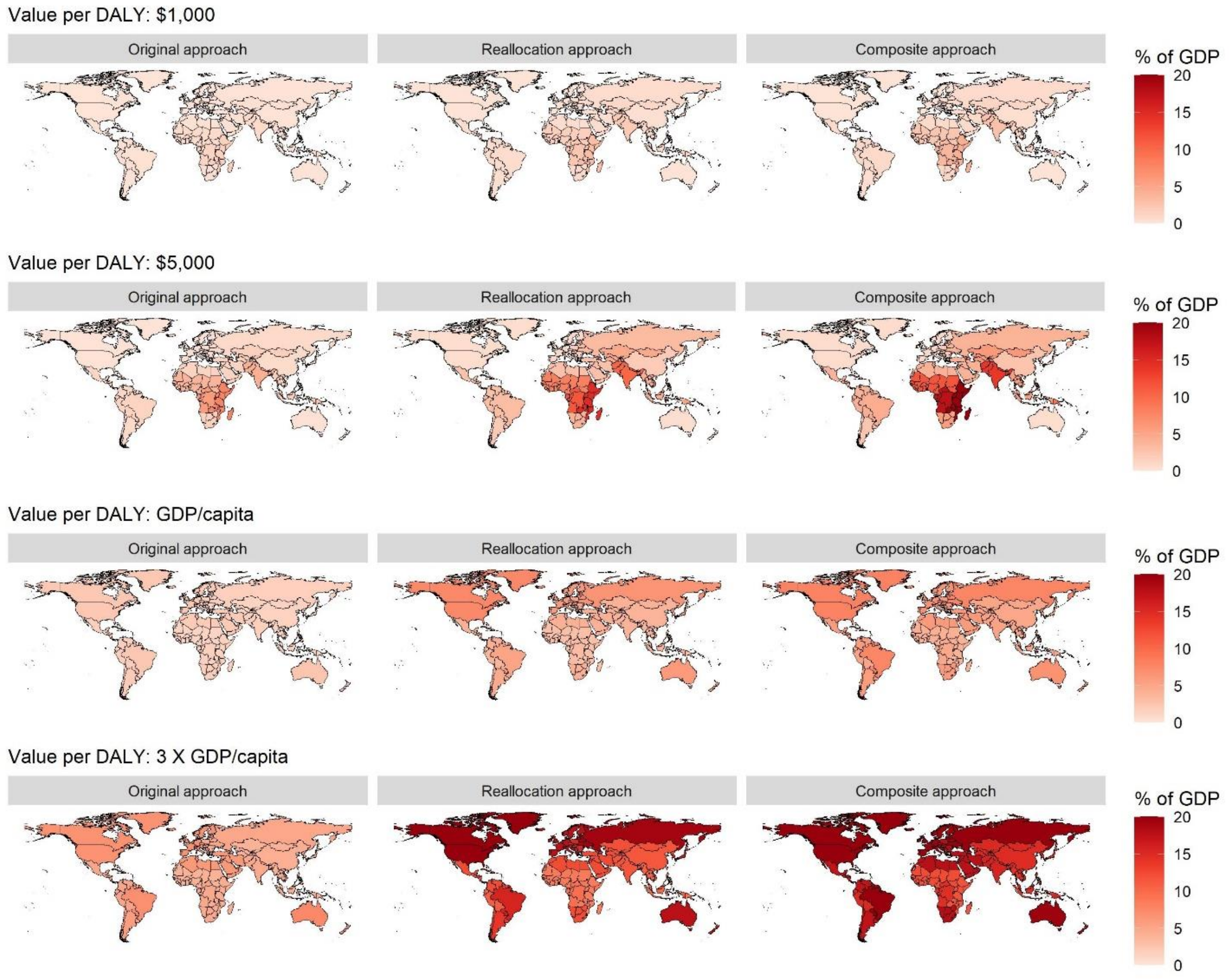

References

1 Bloom DE, Cafiero ET, Jané-Llopis E, *et al.* The Global Economic Burden of Non-communicable Diseases. Geneva, Switzerland: World Economic Forum, 2011 http://www3.weforum.org/docs/WEF_Harvard_HE_GlobalEconomicBurdenNonCommunicableDiseases_2011.pdf (accessed March 11, 2019).

2 Global Burden of Disease Collaborative Network. Global Burden of Disease Study 2019 (GBD 2019) Cause, REI, and Location Hierarchies. 2020. DOI:106·069/Q0YC-CR46.
